# Intelligence

**Published:** 1830-11

**Authors:** 


### INTELLIGENCE.

*Prize of* 1,100*l. offered by the Russian Government for the best Essay on Cholera Morbus.*

*To the Medical Faculty in England.*

CHOLERA MORBUS. The epidemic disorder known under this name has of late years committed the most frightful havoc among various nations in Asia. Within the last fifteen months this awful scourge of human life has made its appearance in several provinces of the Russian empire with undiminished severity. No medical work hitherto published on this fatal malady has been found satisfactory, nor have the suggestions contained in such works succeeded in arresting its devastating course, which, on the contrary, becomes every day more extensive, and seems to threaten the whole of Europe.

Moved by a deep feeling of humanity, the imperial government of Russia deem it right to call the attention of the medical profession in Russia, Germany, Hungary, Italy, England, Sweden, and Denmark to this vitally important subject, and to propose to them to forward to the said government treatises on the cholera morbus, which shall embrace the following points:

1st. A clear and detailed account of the nature of the disease.

2d. A statement of the causes which give rise to it.

3d. A description of its mode of propagation.

4th. A demonstration, by the means of exact and faithful experiments, of its being communicable from one individual to another, if such be the fact.

5th. An indication of the measures to be adopted for self-preservation against its contagion, should the disease prove contagious; and

6th. Of the means best calculated to ensure a recovery.

Such treatises, or essays, may be written in the Russian, Latin, German, English, or Italian languages, and directed to the "Conseil de Médecine" (Medical Board) at St. Petersburg, until the 1st (13) September, 1831. The name of the author is to be forwarded in a separate envelope.

The author of the best treatise, who shall have fully complied with all the above conditions, will be rewarded, by the imperial government of Russia, with a sum of 25,000 roubles in bank paper, amounting to about 1,100 pounds sterling.

This is distributed to the members of the Faculty, with the outside signature of Chevalier Benkbousen, the consul general, who, of course, stamps with authority the document, and becomes necessarily a guarantee for the execution of the promise.

---

The Westminster Medical Society has commenced its meetings for the session, and Dr. Alexander Thomson has, alas! commenced his speeches.

### METEOROLOGICAL JOURNAL,

By Messrs. Harris and Co. Mathematical Instrument Makers, 50, High Holborn.

| | Moon. | Rain gauge. | Thermom. | | | Barometer. | | DeLuc's Hygrom. | | Winds. | | Atmospheric Variations. | | |
|---|---|---|---|---|---|---|---|---|---|---|---|---|---|---|
| | | | 9 a.m. | max. | min. | 9 a.m. | 10p.m. | 9 a.m. | 10p.m. | 9 a.m. | 10p.m. | 9 a.m. | 2 p.m. | 10p.m. |
| Sept. 20 | | | 59 | 61 | 50 | 29.47 | 29.42 | 57 | 56 | SW | SSW | Fine | Fine | Cloudy |
| 21 | | .55 | 52 | 55 | 44 | .15 | .13 | 56 | 57 | S | WSW | Rain | Fine | Fine |
| 22 | | .13 | 52 | 58 | 50 | .37 | .60 | 57 | 58 | WSW | WSW | Fine | —— | —— |
| 23 | | | 57 | 60 | 48 | .30 | .47 | 59 | 58 | S | WSW | Cloudy | Fine | Fine |
| 24 | | .17 | 54 | 59 | 48 | .53 | .43 | 56 | 55 | WSW | WSW | Fine | —— | —— |
| 25 | ☽ | | 55 | 58 | 45 | .60 | .76 | 55 | 54 | WSW | W | —— | Sho'ry | —— |
| 26 | | | 52 | 59 | 49 | .98 | 30.20 | 54 | 55 | NW | WNW | —— | Fine | —— |
| 27 | | | 54 | 62 | 55 | 30.23 | .21 | 57 | 58 | W | SW | Cloudy | —— | Cloudy |
| 28 | | | 58 | 61 | 50 | .11 | 29.98 | 58 | 57 | SW | SW | Cloudy | —— | Fine |
| 29 | | | 53 | 58 | 46 | 29.88 | .88 | 57 | 57 | WNW | NW | Cloudy | —— | —— |
| 30 | | | 51 | 56 | 46 | .94 | .91 | 56 | 56 | NW | SSW | Cloudy | —— | —— |
| Oct. 1 | | | 53 | 57 | 55 | .95 | .95 | 55 | 57 | SW | NE | Fine | —— | —— |
| 2 | ☉ | | 61 | 64 | 57 | .96 | .90 | 58 | 58 | SW | SW | —— | —— | Rain |
| 3 | | .43 | 60 | 62 | 54 | .94 | 30.00 | 60 | 60 | SW | WSW | —— | Cloudy | Fine |
| 4 | | | 59 | 62 | 45 | 30.02 | .16 | 61 | 58 | WSW | N | Cloudy | Fine | —— |
| 5 | | | 56 | 60 | 47 | .24 | .25 | 56 | 56 | NNW | NNW | Fine | —— | —— |
| 6 | | | 52 | 59 | 48 | .23 | .21 | 57 | 58 | NW | NW | —— | Cloudy | —— |
| 7 | | | 54 | 57 | 51 | .22 | .25 | 58 | 59 | NW | NW | Cloudy | Sleet | Cloudy |
| 8 | ☾ | | 56 | 61 | 55 | .30 | .31 | 59 | 58 | NW | NW | Fine | Fine | —— |
| 9 | | | 57 | 59 | 52 | .35 | .35 | 57 | 56 | NNW | NNE | Cloudy | Fine | Cloudy |
| 10 | | | 56 | 58 | 47 | .35 | .31 | 56 | 56 | NNE | NE | Fine | —— | Fine |
| 11 | | | 51 | 55 | 48 | .29 | .19 | 57 | 57 | ESE | ENE | —— | —— | —— |
| 12 | | | 54 | 58 | 45 | .19 | .19 | 57 | 59 | NE | ENE | Cloudy | —— | Sleet |
| 13 | | | 52 | 55 | 47 | .21 | .21 | 59 | 57 | ENE | ESE | Fine | Fine | Fine |
| 14 | | | 52 | 54 | 42 | .14 | .11 | 58 | 56 | ESE | ESE | —— | —— | —— |
| 15 | | | 48 | 51 | 42 | .07 | .07 | 55 | 57 | SE | ESE | —— | —— | —— |
| 16 | ● | | 50 | 54 | 45 | .12 | .16 | 57 | 57 | SE | ESE | —— | —— | —— |
| 17 | | | 49 | 53 | 44 | .19 | .21 | 57 | 57 | SE | SE | Foggy | —— | —— |
| 18 | | | 50 | 56 | 49 | .13 | .01 | 59 | 60 | SE | SE | Fine | —— | Cloudy |
| 19 | | .38 | 55 | 61 | 55 | 29.89 | 29.83 | 60 | 62 | SE | S | Sho'ry | —— | Sho'ry |

The quantity of Rain fallen in September was 2 inches and 92.100ths.

